# Long-Term Mortality in Critically Ill Tracheostomized Patients Based on Home Mechanical Ventilation at Discharge

**DOI:** 10.3390/jpm11121257

**Published:** 2021-11-25

**Authors:** Won-Young Kim, Moon Seong Baek

**Affiliations:** Department of Internal Medicine, Chung-Ang University Hospital, Chung-Ang University College of Medicine, Seoul 06973, Korea; wykim81@cau.ac.kr

**Keywords:** tracheostomy, mechanical ventilators, ventilator weaning, mortality

## Abstract

Data regarding the long-term outcomes for tracheostomized patients receiving home mechanical ventilation (HMV) are limited. We aimed to determine the 1-year mortality rate for critically ill tracheostomized patients with and without HMV. Data of tracheostomized patients between 1 January 2015 and 31 December 2019 were analyzed. A Kaplan-Meier analysis was performed to assess the survival curve of the patients. Among the 124 tracheostomized patients, 102 (82.3%) were weaned from mechanical ventilation (MV), and 22 (17.7%) required HMV at discharge. The overall 1-year mortality rate was 47.6%, and HMV group had a significantly higher 1-year mortality rate than those weaned from MV (41.2% vs. 77.3%, *p* = 0.002). In the Cox proportional hazards regression, BMI (HR 0.913 [95% CI 0.850–0.980], *p* = 0.012), Sequential Organ Failure Assessment (SOFA) score (HR 1.114 [95% CI 1.040–1.193], *p* = 0.002), transfer to a nursing facility (HR 5.055 [95% CI 1.558–16.400], *p* = 0.007), and HMV at discharge (HR 1.930 [95% CI 1.082–3.444], *p* = 0.026) were significantly associated with 1-year mortality. Critically ill tracheostomized patients with HMV at discharge had a significantly higher 1-year mortality rate than those weaned from MV. Low BMI, high SOFA score, transfer to a nursing facility, and HMV at discharge were significantly associated with 1-year mortality.

## 1. Introduction

Home mechanical ventilation (HMV) is used in patients with chronic respiratory failure, and the prevalence of HMV is increasing worldwide [1,2]. Neuromuscular disease and chronic obstructive pulmonary disease are common indications for HMV, and HMV via a mask is the primary interface that has been adopted for these patients. Although the percentage of HMV via tracheostomy users is very low in Western countries (3.1–18%) [1], the rate in Korea is reported to be as high as 63%, according to a nationwide study [3]. A tracheostomy is a procedure that is performed for critically ill patients who are expected to require prolonged mechanical ventilation (MV) [4]. Despite undergoing a tracheostomy, however, patients who experience difficulty weaning off MV require HMV for prolonged ventilation [5].

Critical care is a limited resource, and occupancy of intensive care unit (ICU) beds for the purpose of prolonged weaning from MV leads to ICU overcrowding. In an observational study conducted at 55 ICUs, Li et al. reported that one-fifth of the ICU beds were occupied by patients undergoing MV weaning for an extended period of time [6]. To avoid ICU overcrowding, patients requiring prolonged weaning can be transferred to respiratory intermediate care units outside the ICU or specialized regional weaning centers [5]. However, there are no such facilities in Korea, and patients who require prolonged weaning are often transferred to general wards with HMV. If HMV weaning fails, patients are transferred to nursing facilities or are hospitalized for an extended period of time. Lee et al. reported that among patients who were unsuccessful in weaning off HMV, only 13% were discharged home, whereas the majority (56%) were discharged to other facilities [7]. However, data on these patients are very scarce, especially regarding long-term survival.

It is known that patients who receive MV for more than 14 days have a lower long-term survival rate than those who do not [8,9,10]. Although weaning failure from MV can have a negative impact on the long-term outcomes of patients, previous studies have not found it to be a remarkable feature for prolonged MV (ProVent study) [11,12]. Accordingly, we wanted to determine the long-term mortality rate among tracheostomized patients according to subsequent HMV. We aimed to determine the 1-year mortality of critically ill patients who received a tracheostomy with or without subsequent HMV. Furthermore, we investigated the risk factors for 1-year mortality in these patients.

## 2. Materials and Methods

### 2.1. Study Design and Patients

This single-center retrospective study was conducted at a tertiary university-affiliated hospital in Korea. Critically ill adult patients, including patients with cardiac and cerebrovascular disease, are managed in the 28 beds of the medical ICU of this hospital. Consecutive patients who were mechanically ventilated between 1 January 2015 and 31 December 2019 were reviewed for this study. The electronic medical records of patients aged 18 years and older who received a tracheostomy were analyzed. Patients were excluded if they were discharged or dead within 48 h of hospitalization, did not receive a tracheostomy, or received a tracheostomy due to neuromuscular disease or oropharyngeal cancer. Patients with hopeless discharge or death were also excluded.

Based on guidelines for MV weaning, the need for tracheostomy was determined by the failure of a spontaneous breathing trial or the need for reintubation within 48 h following extubation [12]. We generally recommended tracheostomy to the patients or their surrogates within two weeks of intubation. HMV was provided to patients who were unable to wean from MV, despite having a tracheostomy. Therefore, we classified the tracheostomized patients into the HMV and no HMV groups at hospital discharge.

This study was approved by the Institutional Review Board of Chung-Ang University Hospital (2107-015-19374). The requirement for informed consent was waived due to the retrospective nature of the study.

### 2.2. Data Collection and Definitions

We obtained the following data from the electronic medical records of patients who received a tracheostomy during the study period: age, sex, body mass index (BMI), residency before admission (nursing facility or home), bedridden status, tube feeding before admission, Sequential Organ Failure Assessment (SOFA) score, ProVent 14 score [10], Charlson Comorbidity Index, comorbidities, type of admission (emergency room or outpatient), reason for ICU admission (sepsis/septic shock, pneumonia, cardiovascular disease, acute respiratory distress syndrome/acute respiratory failure, post-cardiac arrest care, or other), type of tracheostomy (surgical tracheostomy vs. percutaneous dilatational tracheostomy), initial vital signs and PaO_2_/FiO_2_, time from MV to tracheostomy, vasopressor, continuous renal replacement therapy, neuromuscular blocker, laboratory findings, discharge location (nursing facility or home), tube feeding at discharge, and tracheostomy tube decannulation. The primary outcome was 1-year mortality, and the secondary outcomes were 3-month and 6-month mortalities, length of hospital stay, duration of ICU stay, duration of MV, and duration of MV and HMV.

The Provent 14 score is a simplified prognostic scoring system that was developed to predict the 1-year mortality rate for patients requiring prolonged MV [10]. Five clinical variables, including age, platelet count, vasopressor requirement, hemodialysis requirement, and non-trauma admission were measured on day 14 of MV.

### 2.3. Statistical Analysis

The categorical variables are expressed as numbers (percentages), and the continuous variables are expressed as medians (interquartile ranges). Pearson’s chi-square test or Fisher’s exact test were used for the categorical data, and the Mann-Whitney U test was used to compare the continuous variables. A Kaplan-Meier analysis was performed to assess the survival curve of the MV patients who received a tracheostomy. Univariate and multivariable Cox proportional hazards regression analyses were performed to determine the independent factors of 1-year mortality. Significant variables from the univariate analyses (*p* < 0.05) and clinically significant variables were included in the multivariable analysis. Kaplan-Meier curves for cumulative survival were created and compared using a log-rank test. All the statistical analyses were performed using the Statistical Package for the Social Sciences version 26.0 (IBM Corporation, Armonk, NY, USA), and *p* values < 0.05 were considered statistically significant.

## 3. Results

### 3.1. Patient Characteristics

**Figure 1:**
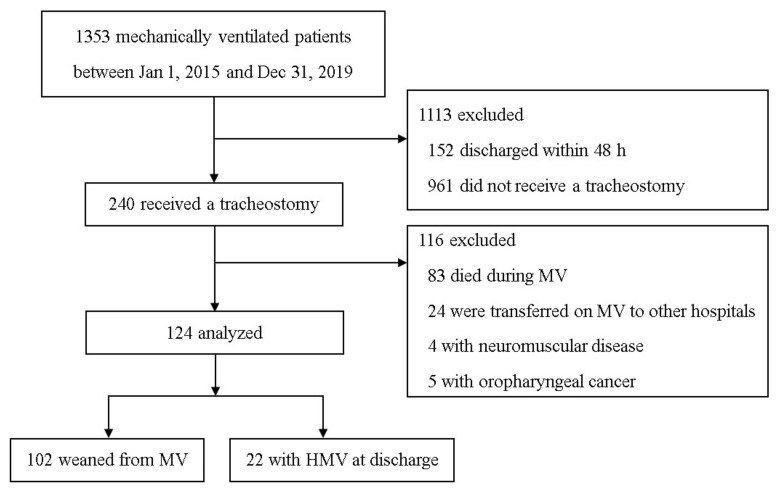
Patient flowchart. MV, mechanical ventilation; HMV, home mechanical ventilation.

During the study period, 1353 patients underwent MV in the medical ICU (Figure 1). A tracheostomy was performed for 240 (17.7%) of these patients. Patients who were discharged within 48 h of ICU admission (*n* = 152), did not receive a tracheostomy (*n* = 961), had neuromuscular disease (*n* = 4) or oropharyngeal cancer (*n* = 5), and those who experienced hopeless discharge or in-hospital death (*n* = 107) were excluded. Of the remaining 124 patients, 102 (82.3%) were weaned off MV, and 22 (17.7%) required HMV at discharge.

The characteristics of the MV patients who received a tracheostomy are presented in Table 1. The median MV patient age was 77 years (IQR: 68–82), and the median BMI was 20.8 kg/m^2^ (IQR: 18.4–24.4). Before admission, 23.4% of patients resided in a nursing home or hospital. The median SOFA score was 8.5 (IQR: 6–11), and the median ProVent 14 score was 2 (IQR: 2–2). Pneumonia (33.9%) and post-cardiac arrest care (28.2%) were common reasons for ICU admission among the tracheostomy patients. The tracheostomies were performed 13 days (IQR: 8–17) after MV initiation.

Compared with the no HMV group, the HMV group had a lower BMI (20.8 kg/m^2^ [IQR: 18.5–24.3] vs. 20.5 kg/m^2^ [IQR: 17.7–24.5], *p* = 0.014), higher SOFA score (8 [IQR: 5–11] vs. 9 [IQR: 7–11], *p* = 0.014), higher incidence of chronic lung disease (5.9% vs. 27.3%, *p* = 0.007), and higher albumin level (3.0 g/dL [IQR: 2.5–3.5] vs. 3.2 g/dL [IQR: 2.8–3.4], *p* = 0.029). At hospital discharge, the tracheostomy tube was decannulated for 24.5% of patients in the no HMV group. All patients with HMV were transferred to nursing homes or hospitals, and each received a feeding tube at discharge. Of the no HMV group, 23.5% (*n* = 24) discharged to home, and 76.5% (*n* = 78) were transferred to nursing facilities. The ICU stay duration tended to be longer for the HMV group than for the no HMV group, although the difference was not statistically significant (52 days [IQR: 31–67] vs. 26 days [IQR: 18–43], *p* = 0.228). The total MV duration (MV and HMV duration) was significantly longer for the HMV group (75 days [IQR: 52–102] vs. 19 days [IQR: 12–29], *p* = 0.034). 

### 3.2. Outcomes of Patients Who Received a Tracheostomy

**Figure 2:**
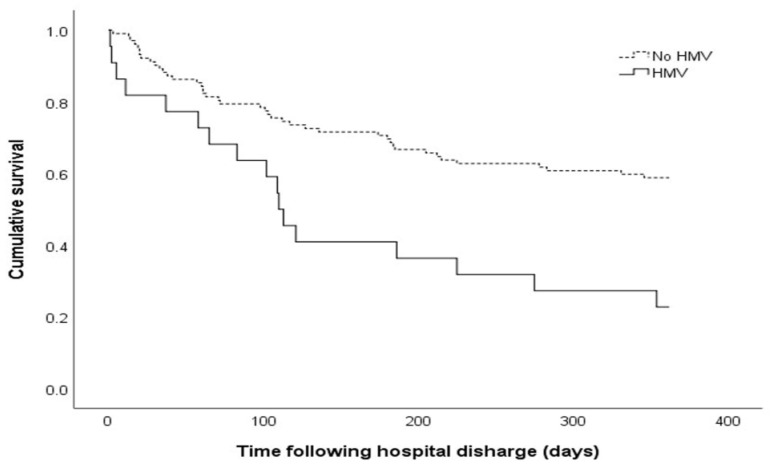
The Kaplan-Meier curve of the one-year survival plots comparing the no HMV group and the HMV group. The one-year survival rates were significantly lower for the HMV group than for the no HMV group (22.7% vs. 58.8%, *p* value = 0.001, Log Rank (Mantel-Cox)). HMV, home mechanical ventilation.

The overall 1-year mortality rate was 47.6%, and the HMV group had a significantly higher 1-year mortality rate than the no HMV group (77.3% vs. 41.2%, respectively; *p* = 0.002) (Table 2 and Figure 2). The six-month mortality rate was higher for the HMV group (59.1% vs. 30.4%, *p* = 0.014). The ICU stay duration tended to be longer for the HMV group than for the no HMV group, although the difference was not statistically significant (52 days [IQR: 31–67] vs. 26 days [IQR: 18–43], *p* = 0.228). The total MV duration (MV and HMV duration) was significantly longer for the HMV group (75 days [IQR: 52–102] vs. 19 days [IQR: 12–29], *p* = 0.034).

### 3.3. Predictive Factors for 1-Year Mortality

The comparison between patients who received a tracheostomy and patients with 1-year mortality is presented in Table S1. The patients with 1-year mortality had a lower BMI (20.2 kg/m^2^ [17.9–23.4] vs. 22.3 kg/m^2^ [IQR: 19.5–24.9], *p* = 0.014), higher SOFA score (9 [IQR: 7–12] vs. 7 [IQR: 5–10], *p* = 0.014), higher incidence of vasopressor use (61.0% vs. 41.5%, *p* = 0.030), lower albumin level (2.8 g/dL [IQR: 2.4–3.3] vs. 3.2 g/dL [IQR: 2.8–3.6], *p* = 0.029), higher transfer to a nursing facility incidence (94.9% vs. 67.7%, *p* < 0.001), higher feeding tube rate at discharge (91.5% vs. 61.5%, *p* < 0.001), and a lower tracheostomy tube decannulation incidence (10.2% vs. 29.2%, *p* = 0.008) compared the 1-year survivors.

The results of the multivariable Cox proportional hazards analysis for the predictors of 1-year mortality are shown in Table 3. In this analysis, BMI (HR 0.913 [95% CI 0.850–0.980], *p* = 0.012), SOFA score (HR 1.114 [95% CI 1.040–1.193], *p* = 0.002), transfer to a nursing facility (HR 5.055 [95% CI 1.558–16.400], *p* = 0.007), and HMV at discharge (HR 1.930 [95% CI 1.082–3.444], *p* = 0.026) were significantly associated with 1-year mortality.

## 4. Discussion

We determined the 1-year mortality rates for MV patients who received a tracheostomy with and without HMV at discharge, as well as the risk factors associated with 1-year mortality for these patients. In our older age study cohort, the overall 1-year mortality rate was 47.6%. However, patients who were discharged with HMV had a significantly higher 1-year mortality rate (77.3%), compared with the no HMV group (41.2%). Low BMI, higher SOFA score, transfer to a nursing facility, and HMV at discharge were significantly associated with 1-year mortality.

For patients who received prolonged MV, long-term mortality was affected by age, ventilator weaning failure, and vasopressor or hemodialysis requirement [13]. Age was shown to be particularly important. The 1-year mortality rate has been reported to be approximately 45% in studies that included patients under the median age of 70 years [14,15]. However, Cohen et al. reported that the 1-year mortality rates for tracheostomized patients >85 years of age and <85 years of age were 82% and 66%, respectively [16]. The results of these studies indicate that age has a significant effect on long-term mortality for tracheostomized patients. Our study also included an elderly study population with a median age of 77 years, and the 1-year mortality rate was significantly different depending on the ventilator weaning success (77% vs. 41%). According to another study by Depuydt et al., the 1-year mortality rate was not significantly different between the ventilator-dependent and weaned tracheostomized patients with a median age of approximately 60 years (37% vs. 41%) [17]. Age is an important prognostic factor for the 1-year mortality of tracheostomized patients; however, ventilator weaning failure is crucial for older tracheostomized patients.

There are limited resources including respiratory intermediate care units or specialized centers for prolonged weaning, and patients who require prolonged weaning are often transferred to general wards with HMV. If HMV weaning fails, patients are transferred to nursing facilities or are hospitalized for an extended period of time. Lee et al. reported that among patients who were unsuccessful in weaning off HMV, only 13% were discharged home, while the majority (56%) were discharged to other facilities [18]. There is a possibility that tracheotomized patients do not receive adequate assistance at home. However, a transfer to a nursing facility was associated with a higher 1-year mortality than discharge to home. This may be attributed to the severity of the patient’s condition at the time of discharge. Severely ill patients were more likely to have been transferred to a nursing facility rather than home; therefore, the nursing facility may have been the risk factor for the higher mortality. In addition, healthcare providers who are skilled at airway management are essential because complications associated with tracheostomies, such as accidental decannulation, are very fatal [19]. However, controlling such emergencies in the nursing facilities can be challenging. In the absence of specialized respiratory care units, continuous education on tracheostomy care and HMV for medical staff in nursing facilities is necessary. Furthermore, tracheostomized patients should receive appropriate treatment through the organizational relationships between the nursing facilities and tertiary hospitals.

Recent nationwide cohort studies that included individuals >65 years of age in Korea have reported that a low BMI is associated with an increased risk of mortality but a high BMI was not [20,21]. Lee et al., reported that the hazard ratio of the BMI < 23 kg/m^2^ group increased significantly compared with those in the BMI = 23–25 kg/m^2^ group. However, the hazard ratio of the BMI > 25 kg/m^2^ group did not increase significantly compared with that of the BMI = 23–25 kg/m^2^ group [21]. A low BMI is known to be associated with malnutrition or sarcopenia [22,23,24]. Malnutrition status [22,23] and sarcopenia [25] are associated with an increased risk of mortality, and malnutrition status is also a significant risk factor for sarcopenia [24]. Our results are consistent with those of these previous studies, as they indicate that a low BMI affects the long-term mortality of critically ill tracheostomized patients. Therefore, these patients should be carefully observed for muscle loss and nutrition status.

Several scoring systems have been suggested to be capable of predicting the long-term mortality of patients receiving prolonged MV [10,11]. The ProVent score was developed to predict the 1-year mortality for patients requiring prolonged MV using variables measured on day 21 of the MV. Carson et al. found that the AUC for the ProVent 21 score was 0.79 [11]. A subsequent ProVent 14 score validation study used variables measured on day 14 of the MV, and the AUC was 0.78 [10]. However, these studies were conducted with patients with a mean age of 53~55 years and a 1-year mortality rate of 45~48%. In the study cohort, age, platelets, vasopressors, and hemodialysis were risk factors for 1-year mortality. Unlike those studies, low BMI, higher SOFA score, transfer to a nursing facility, and HMV at discharge were prognostic factors in our cohort. Therefore, additional prospective studies involving a larger number of patients are needed to address the long-term mortality prediction model using the risk factors presented in the current study.

This study has several limitations. First, this was a single center, retrospective study design, therefore, data for do-not-resuscitate orders or indications of HMV could not be obtained. In our study, 81% of the overall patients (100% of patients in the HMV group) were transferred to a nursing home or hospital. Transfer to a nursing facility is a significant risk factor for long-term mortality. In Korea, many older patients are discharged with HMV after ICU care ends because it is difficult to wean them off MV. Therefore, there is the possibility that HMV could be utilized as a palliative chronic respiratory failure treatment for end-of-life care [2,26]. Second, unfortunately, we did not present clear reasons for tracheostomy insertion, which may make our study population seem heterogeneous. However, we only enrolled medical ICU patients, and the reasons for ICU admission were mostly pneumonia, sepsis, or acute respiratory distress syndrome. Furthermore, we excluded patients who had neurological disorders or oropharyngeal tumors. We tried to enroll a homogenous cohort comprising critically ill patients who were mechanically ventilated and received tracheostomies. Third, this study comprised a relatively small number of patients; therefore, our sample size may not have been large enough to make the results generalizable. As critical care medicine advances, HMV use is increasing for survivors after ICU care ends. Therefore, further prospective studies that include a larger number of patients are needed to identify the associations between HMV and long-term mortality for older critically ill patients.

## 5. Conclusions

The critically ill tracheostomized patients with HMV at discharge had a significantly higher 1-year mortality rate than patients who were weaned from MV. Low BMI, high SOFA score, transfer to a nursing facility, and HMV at discharge were significantly associated with 1-year mortality.

## Figures and Tables

**Table 1:** Baseline characteristics of the mechanically ventilated patients who received a tracheostomy.

Variables	Total (*n* = 124)	No HMV (*n* = 102)	HMV (*n* = 22)	*p* Value
Age (years)	77 (68–82)	76 (69–82)	78 (66–83)	0.094
Male (%)	69 (55.6)	55 (53.9)	14 (63.6)	0.405
Body mass index (kg/m^2^)	20.8 (18.4–24.4)	20.8 (18.5–24.3)	20.5 (17.7–24.5)	0.014
Before admission (%)				
Nursing home or hospital	29 (23.4)	26 (25.5)	3 (13.6)	0.234
Bedridden status	29 (23.4)	26 (25.5)	3 (13.6)	0.234
Tube feeding	29 (23.4)	24 (23.5)	5 (22.7)	0.936
SOFA score	8.5 (6–11)	8 (5–11)	9 (7–11)	0.014
ProVent 14 score	2 (2–2)	2 (2–2)	2 (2–3)	0.096
Charlson Comorbidity Index	4 (3–5)	4 (3–5)	3.5 (3–5)	0.383
Comorbidities (%)				
Diabetes	11 (8.9)	7 (6.9)	4 (18.2)	0.105
Hypertension	12 (9.7)	9 (8.8)	3 (13.6)	0.445
Chronic lung disease	12 (9.7)	6 (5.9)	6 (27.3)	0.007
Chronic kidney disease	6 (4.8)	3 (2.9)	3 (13.6)	0.068
Chronic liver disease	2 (1.6)	2 (2.0)	0 (0.0)	1.000
Cardiovascular disorder	24 (19.4)	19 (18.6)	5 (22.7)	0.766
Neurological disorder	30 (24.2)	25 (24.5)	5 (22.7)	1.000
Malignancy	6 (4.8)	6 (5.9)	0 (0.0)	0.590
Admission via ER (%)	109 (87.9)	92 (90.2)	17 (77.3)	0.141
Reason for ICU admission (%)				0.163
Sepsis/septic shock	18 (14.5)	16 (15.7)	2 (9.1)	
Pneumonia	42 (33.9)	38 (37.3)	4 (18.2)	
Cardiovascular disease	5 (4.0)	5 (4.9)	0 (0.0)	
ARDS/acute respiratory failure	13 (10.5)	10 (9.8)	3 (13.6)	
Post-cardiac arrest care	35 (28.2)	26 (25.5)	9 (40.9)	
Other	11 (8.9)	7 (6.9)	4 (18.2)	
Tracheostomy type (%)				0.184
Surgical tracheostomy	91 (73.4)	72 (70.6)	19 (86.4)	
Percutaneous dilatational tracheostomy	33 (26.6)	30 (29.4)	3 (13.6)	
Initial vital signs				
Systolic blood pressure (mmHg)	94 (87–107)	94 (87–106)	95.5 (90–110)	0.739
Diastolic blood pressure (mmHg)	54 (48–60)	54 (48–59)	54 (46–65)	0.622
Heart rate (/min)	94 (82–110)	94 (80–106)	108 (88–126)	0.499
Respiratory rate (/min)	22.5 (20–26)	22 (20–26)	24 (19–28)	0.725
Body temperature (°C)	36.7 (36.4–37.2)	36.7 (36.5–37.2)	36.7 (36.2–37.2)	0.068
Oxygen saturation (%)	97 (95–99)	97 (95–99)	97 (94–99)	0.762
Glasgow coma scale	7 (5–10)	7 (5–10)	7 (5–10)	0.834
PaO_2_/FiO_2_	171 (94–254)	177 (99–258)	153 (74–274)	0.149
Time of MV to tracheostomy (days)	13 (8–17)	13 (8–18)	13 (10–15)	0.272
Vasopressor (%)	63 (50.8)	50 (49.0)	13 (59.1)	0.391
CRRT (%)	18 (14.5)	12 (11.8)	6 (27.3)	0.090
Neuromuscular blocker (%)	10 (8.1)	7 (6.9)	3 (13.6)	0.383
Laboratory findings				
White blood cells (×10^9^/L)	12.4 (8.3–17.0)	13.1 (8.4–16.9)	10.3 (8.3–20.6)	0.296
Platelet (×10^9^/L)	226 (161–308)	225 (162–295)	228 (132–355)	0.647
Albumin (g/dL)	3.0 (2.6–3.5)	3.0 (2.5–3.5)	3.2 (2.8–3.4)	0.029
Creatinine (mg/dL)	0.91 (0.57–1.47)	0.87 (0.55–1.46)	1.04 (0.70–2.25)	0.079
C-reactive protein (mg/dL)	94 (16–182)	87 (17–177)	110 (11–229)	0.074
Lactic acid (mmol/L)	1.9 (1.0–3.5)	1.9 (1.0–3.8)	1.6 (1.1–2.4)	0.371
At discharge (%)				
Nursing home or hospital	100 (80.6)	78 (76.5)	22 (100.0)	0.007
Tube feeding	94 (75.8)	72 (70.6)	22 (100.0)	0.003
Decannulation of tracheostomy tube	25 (20.2)	25 (24.5)	0 (0.0)	0.007
Length of hospital stay (days)	68 (42–106)	72 (42–106)	62 (40–112)	0.136
Duration of ICU stay (days)	29 (21–52)	26 (18–43)	52 (31–67)	0.228
Duration of MV (days)	22 (13–34)	19 (12–29)	33 (26–52)	0.082
Duration of MV and HMV (days)	23 (13–46)	19 (12–29)	75 (52–102)	0.034

The data are shown as the median (IQR) or number (%). MV = mechanical ventilation; HMV = home mechanical ventilation; SOFA = Sequential Organ Failure Assessment; ER = emergency room; ICU = intensive care unit; ARDS = acute respiratory distress syndrome; PaO_2_ = partial pressure of oxygen; FiO_2_ = fraction of inspired oxygen; CRRT = continuous renal replacement therapy.

**Table 2:** Patient outcomes.

Variables	Total (*n* = 124)	No HMV (*n* = 102)	HMV (*n* = 22)	*p* Value
Primary outcome				
1-yr mortality (%)	59 (47.6)	42 (41.2)	17 (77.3)	0.002
Secondary outcomes				
3-m mortality (%)	29 (23.4)	21 (20.6)	8 (36.4)	0.162
6-m mortality (%)	44 (35.5)	31 (30.4)	13 (59.1)	0.014

The data are shown as the median (IQR) or number (%). MV = mechanical ventilation; HMV = home mechanical ventilation; ICU = intensive care unit.

**Table 3:** Univariate and multivariable Cox proportional analyses of the variables associated with 1-year mortality.

Variables	Univariate Analysis		Multivariable Analysis	
	HR (95% CI)	*p* value	HR (95% CI)	*p* value
Age	1.009 (0.989–1.029)	0.388		
Male	1.694 (0.993–2.889)	0.053		
Body mass index	0.917 (0.856–0.982)	0.013	0.913 (0.850–0.980)	0.012
Bedridden status before admission	1.150 (0.640–2.066)	0.641		
Tube feeding before admission	1.314 (0.740–2.332)	0.352		
SOFA score	1.080 (1.011–1.154)	0.023	1.114 (1.040–1.193)	0.002
ProVent 14 score	1.204 (0.881–1.644)	0.244		
Charlson Comorbidity Index	1.115 (0.971–1.279)	0.123		
Vasopressor	1.772 (1.048–2.995)	0.033		
CRRT	2.113 (1.139–3.918)	0.018		
Neuromuscular blocker	1.815 (0.823–4.004)	0.140		
Transferred to a nursing facility	6.156 (1.925–19.684)	0.002	5.055 (1.558–16.400)	0.007
Tube feeding at discharge	4.733 (1.891–11.843)	0.001		
Decannulation of tracheostomy tube	0.362 (0.156–0.843)	0.018		
HMV at discharge	2.560 (1.453–4.510)	0.001	1.930 (1.082–3.444)	0.026
Length of hospital stay	0.997 (0.993–1.001)	0.171		
Duration of ICU stay	1.006 (0.996–1.015)	0.227		
Duration of MV and HMV	1.004 (1.000–1.008)	0.032		
Time from MV to tracheostomy	0.993 (0.977–1.009)	0.361		
PaO_2_/FiO_2_	0.999 (0.997–1.001)	0.315		
Albumin	0.619 (0.406–0.946)	0.027		

The clinical variables entered into the model were age, sex, body mass index, SOFA score, ProVent 14 score, Charlson Comorbidity Index, vasopressor, CRRT, transferred to nursing facilities, tube feeding at discharge, decannulation of tracheostomy tube, HMV at discharge, and albumin. HR = hazard ratio; CI = confidence interval; SOFA = Sequential Organ Failure Assessment; CRRT = continuous renal replacement therapy; HMV = home mechanical ventilation; ICU = intensive care unit; MV = mechanical ventilation; PaO_2_ = partial pressure of oxygen; FiO_2_ = fraction of inspired oxygen.

## Data Availability

The datasets used and analyzed during the current study are available from the corresponding author on reasonable request.

## Supplementary Materials

The following are available online at https://www.mdpi.com/article/10.3390/jpm11121257/s1, Table S1: Comparison between the mechanically ventilated patients who received a tracheostomy according to the 1-yr mortality and 1-yr survival.
