# Supplementary material for: Long-Term Mortality in Critically Ill Tracheostomized Patients Based on Home Mechanical Ventilation at Discharge
Source: J Pers Med. 2021 Nov 25;11(12):1257. doi: 10.3390/jpm11121257 (PMC8706308; doi:10.3390/jpm11121257)
Supplement: Supplementary file 1 [file jpm-11-01257-s001.zip › jpm-1409756-supplementary.pdf]

Supplementary Material

Table S1. Comparison between the mechanically ventilated patients who received a tracheostomy according to the 1-yr mortality and 1-yr survival.

Variables	1-yr survival (*n* = 65)	1-yr mortality (*n* = 59)	*p* value
**Age (years)**	74 (66–82)	79 (71–83)	0.094
**Male (%)**	31 (47.7)	38 (64.4)	0.061
**Body mass index (kg/m²)**	22.3 (19.5–24.9)	20.2 (17.9–23.4)	0.014
**Before admission (%)**			
**Nursing home or hospital**	14 (21.5)	15 (25.4)	0.610
**Bedridden status**	14 (21.5)	15 (25.4)	0.610
**Tube feeding**	13 (20.0)	16 (27.1)	0.350
**SOFA score**	7 (5–10)	9 (7–12)	0.014
**ProVent 14**	2 (2–2)	2 (2–3)	0.096
**Charlson Comorbidity Index**	4 (3–5)	4 (3–5)	0.383
**Comorbidities (%)**			
**Diabetes**	5 (7.7)	6 (10.2)	0.628
**Hypertension**	7 (10.8)	5 (8.5)	0.666
**Chronic lung disease**	6 (9.2)	6 (10.2)	0.860
**Chronic kidney disease**	1 (1.5)	5 (8.5)	0.101
**Chronic liver disease**	1 (1.5)	1 (1.7)	1.000
**Cardiovascular disorder**	14 (21.5)	10 (16.9)	0.518
**Neurological disorder**	15 (23.1)	15 (25.4)	0.761
**Malignancy**	2 (3.1)	4 (6.8)	0.423
**Admission via ER (%)**	60 (92.3)	49 (83.1)	0.114
**Reason for ICU admission (%)**			0.752
**Sepsis/septic shock**	8 (12.3)	10 (16.9)	
**Pneumonia**	23 (35.4)	19 (32.2)	
**Cardiovascular disease**	2 (3.1)	3 (5.1)	
**ARDS/acute respiratory failure**	5 (7.7)	8 (13.6)	
**Post-cardiac arrest care**	21 (32.3)	14 (23.7)	
**Other**	6 (9.2)	5 (8.5)	
**Tracheostomy type (%)**			0.903
**Surgical**	48 (73.8)	43 (72.9)	
**Percutaneous dilatational tracheostomy**	17 (26.2)	16 (27.1)	
**Initial vital signs**			
**Systolic blood pressure (mmHg)**	93 (89–106)	95 (86–110)	0.739
**Diastolic blood pressure (mmHg)**	54 (48–60)	54 (46–62)	0.622
**Heart rate (/min)**	96 (81–112)	92 (82–108)	0.499
**Respiratory rate (/min)**	22 (20–26)	23 (20–27)	0.725
**Body temperature (°C)**	36.8 (36.5–37.5)	36.7 (36.3–37.0)	0.068
**Oxygen saturation (%)**	97 (95–99)	97 (95–99)	0.762
**Glasgow coma scale**	7 (5–10)	7 (5–10)	0.834
**PaO_2/FiO_2**	182 (100–283)	146 (76–249)	0.149
**Time from MV to tracheostomy (days)**	14 (8–18)	12 (8–16)	0.272
**Vasopressor (%)**	27 (41.5)	36 (61.0)	0.030
**CRRT (%)**	5 (7.7)	13 (22.0)	0.024
**Neuromuscular blocker (%)**	3 (4.7)	7 (11.9)	0.193
**Laboratory findings**			
**White blood cells (×10^9/L)**	13.4 (8.8–17.2)	12.0 (8.2–17.1)	0.296
**Platelet (×10^9/L)**	227 (168–305)	226 (148–315)	0.647

**Albumin (g/dL)**	3.2 (2.8–3.6)	2.8 (2.4–3.3)	0.029
**Creatinine (mg/dL)**	0.77 (0.54–1.40)	0.97 (0.64–1.75)	0.079
**C-reactive protein (mg/dL)**	68 (12–160)	108 (37–201)	0.074
**Lactic acid (mmol/L)**	1.7 (0.9–2.9)	1.9 (1.1–3.8)	0.371
**At discharge (%)**			
**Transferred to a nursing home or hospital**	44 (67.7)	56 (94.9)	<0.001
**Tube feeding**	40 (61.5)	54 (91.5)	<0.001
**Decannulation of tracheostomy tube**	19 (29.2)	6 (10.2)	0.008
**Length of hospital stay (days)**	77 (42–107)	59 (37–94)	0.136
**Duration of ICU stay (days)**	27 (18–51)	31 (23–56)	0.228
**Duration of MV (days)**	20 (12–29)	24 (16–40)	0.082
**Duration of MV and HMV (days)**	21 (12–33)	26 (16–64)	0.034

The data are shown as the median (IQR) or number (%).

SOFA=Sequential Organ Failure Assessment; ER=emergency room; ICU=intensive care unit; ARDS=acute respiratory distress syndrome; PaO_2=partial pressure of oxygen; FiO_2=fraction of inspired oxygen; MV=mechanical ventilation; CRRT=continuous renal replacement therapy; and HMV=home mechanical ventilation.
